# *Acanthamoeba castellanii*: Non-Steroidal Anti-Inflammatory Drugs Affect Adhesion, Motility, and Encystment, Suggesting a Link with a gp63-like Protein Candidate

**DOI:** 10.3390/pathogens15030263

**Published:** 2026-03-02

**Authors:** Verónica I. Hernández-Ramírez, Hugo Varela-Rodríguez, Luis Varela-Rodríguez, Francisco Sierra-López, Daniela Eloísa San Juan-Mora, José Daniel Morales-Mora, Daniela Falcón-Navarrete, Carlos Osorio-Trujillo, Jacqueline Ríos-López, Itzel Berenice Rodríguez-Mera, María Maricela Carrasco-Yépez, Patricia Talamás-Rohana

**Affiliations:** 1 Departamento de Infectómica y Patogénesis Molecular, Cinvestav, Ciudad de México 07360, Mexico; vhernandezr@cinvestav.mx (V.I.H.-R.); luck19861990@gmail.com (F.S.-L.); daniel.morales@cinvestav.mx (J.D.M.-M.); jcosorio@cinvestav.mx (C.O.-T.); jacrios176@gmail.com (J.R.-L.); 2 Facultad de Medicina y Ciencias Biomédicas, Universidad Autónoma de Chihuahua, Chihuahua 31125, Mexico; hvrodriguez@uach.mx; 3 Facultad de Ciencias Químicas, Universidad Autónoma de Chihuahua, Chihuahua 31125, Mexico; lvrodriguez@uach.mx; 4 Unidad de Investigación en Salud, Hospital Regional de Alta Especialidad de Ixtapaluca, Ciudad de México 56530, Mexico; 5 Departamento de Genética y Biología Molecular, Cinvestav, Ciudad de México 07360, Mexico; daniela.sanjuan@cinvestav.mx; 6 Unidad de Investigación Interdisciplinaria en Ciencias de la Salud y la Educación, Facultad de Estudios Superiores Iztacala, Universidad Nacional Autónoma de México, Tlalnepantla de Baz 54090, Mexico; daniela.falcon@yahoo.com (D.F.-N.); ib.rodriguez@iztacala.unam.mx (I.B.R.-M.); macaye@iztacala.unam.mx (M.M.C.-Y.)

**Keywords:** *Acanthamoeba castellanii*, gp63-like, leishmanolysin, cyclooxygenase inhibitors, NSAIDs

## Abstract

*Acanthamoeba castellanii*, an opportunistic free-living amoeba, causes severe infections including Acanthamoeba keratitis. This exploratory study evaluated whether three non-steroidal anti-inflammatory drugs (NSAIDs)—acetylsalicylic acid, ibuprofen, and diclofenac (100 µM)—modulate pathogenicity-related processes in *A. castellanii* and explored the involvement of a gp63-like protein during encystment and adhesion. Trophozoites were continuously exposed to each drug and analyzed for adhesion, migration on host-derived discontinuous brain micropatterns, encystment efficiency, and parasite-induced cytoskeletal remodeling in MDCK epithelial cells. In silico docking was performed to assess potential drug–protein interactions. Drug exposure reduced adhesion with maximal inhibition at 60 min. After 1 h, migration decreased by 49%, 64%, and 38%, and encystment was reduced by 50%, 85%, and up to 90%, respectively, in cultures treated with acetylsalicylic acid, ibuprofen, and diclofenac. Co-incubation with untreated trophozoites lowered actin fluorescence to approximately 50%, whereas drug-treated co-cultures preserved fluorescence near control levels. Colocalization analysis showed increased spatial overlap between gp63-like protein and F-actin in cysts (~40%) and migrating trophozoites (~20%) compared with non-stimulated forms (~3.8%). Collectively, these findings suggest that NSAID-sensitive pathways influence host interaction, migration, and encystment in *A. castellanii* and allow for the proposal of gp63-like protein as a putative molecular component of the NSAIDs sensitive pathways.

## 1. Introduction

*Acanthamoeba castellanii* is a ubiquitous free-living amoeba found in soil, water, air, and man-made environments. It is an opportunistic pathogen capable of causing severe human infections, including amoebic keratitis, granulomatous amoebic encephalitis, and disseminated disease affecting the skin and lungs [1,2]. These infections are often difficult to treat and may be fatal, particularly in immunocompromised individuals [3]. Although these disease entities are clinically distinct, they share conserved early pathogenic mechanisms such as adhesion to host tissues, directed migration, cytoskeleton-dependent interactions with host cells, and developmental transitions associated with encystment. These processes precede tissue invasion and cyst persistence, and therefore represent common pathogenic denominators rather than disease-specific traits. Accordingly, the present study does not aim to model a single clinical entity, but rather to examine regulatory pathways underlying core pathogenic behaviors relevant to multiple manifestations of *Acanthamoeba* infection.

The life cycle of *A. castellanii* alternates between a metabolically active trophozoite and a highly resistant cyst. Encystment and excystation are critical for parasite persistence and disease progression [4]. Successful colonization and host tissue invasion depend on regulated signaling pathways controlling parasite–substrate and parasite–host interactions. These pathways coordinate adhesion, motility, and intracellular persistence [5].

Lipid mediators derived from arachidonic acid, including prostanoids, play central roles in inflammation and cellular communication; several protozoan parasites are capable of producing these molecules through cyclooxygenase (COX)-like pathways [6,7,8,9,10,11,12]. In parasitic models such as *Leishmania mexicana*, the surface metalloprotease gp63 (leishmanolysin) has been functionally linked to prostaglandin production, providing a conceptual reference for exploring analogous pathways in other protozoa [13,14]. In *A. castellanii*, evidence suggests the presence of COX-sensitive mechanisms involved in key biological processes, as solubilized trophozoite fractions and extracellular vesicles exhibit COX-like activity, although the molecular identity of the responsible protein(s) remains unresolved [14,15,16]. A gp63-like protein has been identified in *A. castellanii* based on sequence homology and conserved structural motifs characteristic of M8 family zinc-dependent metalloproteases [15,17]. While its direct involvement in prostanoid metabolism has not been demonstrated, this protein represents a putative molecular component potentially associated with NSAID-sensitive pathways.

Nonsteroidal anti-inflammatory drugs (NSAIDs), such as aspirin, ibuprofen, and diclofenac, are classical COX inhibitors widely used to modulate prostanoid-dependent pathways [18]. In protozoan parasites, NSAIDs provide a pharmacological tool to explore the functional relevance of prostanoid-related signaling. In *A. castellanii*, NSAIDs have been reported to suppress encystment, suggesting that COX-like activity contributes to life cycle regulation [19]. However, the molecular basis of these effects remains poorly defined.

In this study, we evaluated the effects of acetylsalicylic acid, ibuprofen, and diclofenac on adhesion, motility, proliferation, and within enriched encysted-form preparations obtained after differentiation of *Acanthamoeba castellanii* and examined the localization and redistribution of a gp63-like protein during trophozoite migration and cyst formation. In parallel, molecular docking and in silico pharmacological analyses were performed to assess the structural plausibility of associations between NSAIDs and the gp63-like protein.

Previous studies have demonstrated that non-steroidal anti-inflammatory drugs affect encystment and growth-related processes in *Acanthamoeba* [19]. In the present study, we build upon these observations by applying an integrated, process-oriented approach to further explore how NSAID-sensitive pathways relate to early pathogenic behaviors under host-derived experimental conditions.

By combining quantitative analyses of trophozoite adhesion and migration on brain tissue-derived discontinuous micropatterns (BTDM), parasite-induced actin cytoskeletal remodeling in host cells, and the spatial redistribution of a gp63-like protein during migration and encystment, this study expands the experimental context in which NSAID-associated effects on *Acanthamoeba* biology have been examined. Rather than defining a specific molecular target or mechanism, our results suggest that NSAID exposure is accompanied by coordinated changes in interaction- and differentiation-linked processes, providing a basis for further investigation in future biochemical and genetic studies.

## 2. Materials and Methods

### 2.1. Drugs

Commercial pharmaceutical formulations were used within an exploratory experimental framework to assess NSAID-sensitive biological responses in *Acanthamoeba castellanii*. Pharmacological treatments included acetylsalicylic acid (500 mg effervescent tablets, Aspirin^®^, Bayer), sodium diclofenac (75 mg/3 mL injectable solution, AMSA), and ibuprofen (600 mg oral capsules, Gelubrin^®^). Stock solutions (“mother solutions”) were prepared at 10 mM as follows: acetylsalicylic acid tablets were dissolved in sterile water; diclofenac ampoules were directly diluted in the appropriate culture medium—either Chang medium for assays with trophozoites or cysts, or DMEM for co-culture experiments with trophozoites and MDCK cells; ibuprofen capsules were processed by extracting the gel content and dissolving it in DMSO, followed by dilution in Chang medium or DMEM as required. Working solutions were prepared by a 1:100 dilution of the stock to reach a final concentration of 100 μM. Vehicle controls were included for all treatments, using the same solvents and dilution procedures without the active drug. For diclofenac and acetylsalicylic acid, the vehicles corresponded to the respective culture media, while for ibuprofen, the final DMSO concentration in cultures did not exceed 0.1% (*v*/*v*). Amoebae were not washed after treatment, maintaining continuous exposure to NSAIDs to allow evaluation of cumulative effects. Incubation conditions were tailored to each experimental assay. Adhesion assays: 1 h exposure. Migration/displacement assays (on BTDM): 1 h exposure. Encystment assays: continuous exposure for 7 days to cover cysts development. Growth/proliferation assays: continuous exposure for 24–72 h (Table S1).

### 2.2. Amoebic Cultivation Conditions

*Acanthamoeba castellanii* (strain ATCC 30010/Neff) trophozoites were cultured at 25 °C in Chang’s medium [20] and harvested during the logarithmic growth phase (48–72 h).

### 2.3. Growth Curves

Dose–response curves were generated using different concentrations of NSAIDs over a 24 h period to determine non-cytotoxic concentrations for subsequent assays. A final concentration of 100 µM was selected for all drugs, as this concentration preserved the viability of trophozoites at approximately 93%, as determined by trypan blue exclusion. For proliferation assays, 2.5 × 10^5^ trophozoites were seeded in sterile 10 mL tubes containing Chang’s medium and incubated with NSAIDs, individually, at a final concentration of 100 µM for 24, 48, and 72 h. After incubation, the medium was removed to recover non-adherent cells, and fresh cold medium was added. Tubes containing both decanted and fresh medium were incubated on ice for 10 min and centrifuged at 300× *g* for 20 min at 4 °C. Pellets were resuspended in 500 µL PBS, and 10 µL aliquots were counted using a Neubauer chamber under an inverted optical microscope (Nikon Diaphot, Nikon Corporation, Tokyo, Japan). Viability was assessed in duplicate using trypan blue. Each experiment was performed in triplicate on three independent occasions.

### 2.4. Adhesion Assay in the Presence of Cyclooxygenase Inhibitors

For adhesion assays, 1 × 10^6^ trophozoites were seeded in 25 cm^2^ culture flasks and grown to approximately 95% confluence. NSAIDs were added, individually, at a final concentration of 100 µM in 30 mL of Chang’s medium. Untreated trophozoites served as controls. Adhesion was evaluated at 5, 15, 30, and 60 min. After incubation, samples were decanted and washed with Chang’s medium at room temperature. Flasks were then incubated with 10 mL of fresh Chang’s medium on ice. The suspension was centrifuged at 200× *g* for 10 min at 4 °C, and the pellet was resuspended in 1 mL of fresh medium. Cell counts were performed using a Neubauer chamber under an inverted optical microscope. Assays were conducted in triplicate across three independent experiments.

### 2.5. Evaluation of Cellular Damage (Cytopathic Effect)

The cytopathic effect induced by *A. castellanii* in the presence or absence of cyclooxygenase inhibitors was evaluated using MDCK cells as reported previously [21]. MDCK cells (0.25 × 10^6^) were cultured on coverslips in Petri dishes using DMEM supplemented with 10% fetal bovine serum (FBS) and maintained at 37 °C in a 5% CO_2_ atmosphere for 48 h. Trophozoites (1 × 10^6^) were then co-cultured with MDCK cells at a defined ratio, and NSAIDs were added, individually, at a final concentration of 100 µM. After 24 h, non-adherent trophozoites were removed, and cells were fixed with 4% paraformaldehyde in PBS for 30 min. Samples were processed for confocal microscopy. Coverslips were incubated with rhodamine-conjugated phalloidin (1:50) to visualize F-actin, counterstained with 4′,6-diamidino-2-fenilindol (DAPI) using Vectashield (Vector Laboratories, Inc. Newark, CA, USA) and examined using a Carl Zeiss LSM 900 confocal microscope (Carl Zeiss GmbH, Oberkochen, Germany) (40× using the software Zen Blue edition). In some cases, a 3 × 3 TileScan was applied. Controls included MDCK cells alone, MDCK cells treated with NSAIDs, and MDCK cells co-cultured with trophozoites without inhibitors. The experiments were performed in triplicate on three separate occasions.

### 2.6. Brain Tissue Discontinuous Micropatterns (BTDM)

To investigate migration-related behavior under biologically relevant conditions, brain tissue-derived discontinuous micropatterns (BTDM) were employed as a host-derived experimental substrate to stimulate and analyze trophozoite adhesion and motility. BTDM does not represent a model of granulomatous amoebic encephalitis or any specific disease entity but, rather, provides a complex microenvironment to interrogate conserved migration-associated and cytoskeletal processes.

Brain tissue from BALB/c mice (6–8 weeks old) was obtained from the frontal plane (~0.3 g). Tissue was washed with PBS (pH 7.4), mechanically disrupted, and suspended in 2 mL PBS. After centrifugation at 400× *g* for 10 min, the supernatant was collected and protein concentration adjusted to 10 µg/µL using the Bradford assay. Protein profiles were verified by 12% SDS-PAGE. BTDM were prepared following a modified protocol [22], achieving a protein density of 1–2.5 µg/cm^2^. Suspensions were distributed on plastic or glass surfaces using bidirectional movements, air-dried, heat-inactivated at 65 °C for 10 min, and UV-sterilized. BTDM formation was confirmed by inverted microscopy and used for trophozoite stimulation.

### 2.7. Monitoring of Trophozoite Migration

After 1 h of stimulation with BTDM, with or without NSAIDs, trophozoite migration was monitored for 60 s using an inverted optical microscope equipped with a 20× objective. Subsequently, trophozoites were incubated with NSAIDs (aspirin, ibuprofen, or diclofenac; 100 µM). Migration speed was calculated using the optical field diameter (1.940 mm) and expressed in µm/s.

### 2.8. Confocal Microscopy of gp63-like Protein and F-Actin

Given that cyclooxygenase inhibitors affected trophozoite adhesion, the association between a gp63-like protein and actin cytoskeleton organization was investigated. Trophozoites incubated in the presence or absence of BTDM were fixed with 4% paraformaldehyde and processed for immunofluorescence analysis. Samples were incubated with a commercial monoclonal anti-*Leishmania major* gp63 antibody (anti-*Leishmania major* monoclonal antibody (Cat. No. CLP005A, (CEDARLANE Laboratories Limited, Burlington, ON, Canada;), which recognizes conserved epitopes within the gp63-family metalloproteases in their native state, followed by appropriate fluorescent secondary antibodies. Filamentous actin was visualized using rhodamine-conjugated phalloidin. Images were acquired using the LSM900 confocal laser scanning microscope (Carl Zeiss Microscopy GmbH, Oberkochen, Germany), and colocalization analyses were performed on representative optical sections using the software Zen Black edition.

### 2.9. Detection of gp63-like Protein by Western Blot

A gp63-like protein was detected in membrane-enriched fractions of *Acanthamoeba castellanii* trophozoites by Western blot analysis. Membrane fractions were prepared as previously described by Sierra-López et al. [16], with minor modifications. Briefly, trophozoites were washed twice with ice-cold PBS and resuspended in hypotonic lysis buffer (10 mM Tris-HCl, pH 7.4) supplemented with a protease inhibitor cocktail. To enhance antigen preservation and stabilize associated molecular complexes, cells were fixed with paraformaldehyde (0.1%) prior to lysis, following an approach reported to maintain antigenicity and protein complex integrity in related experimental systems. Cell disruption was performed by mechanical homogenization on ice, and unbroken cells and nuclei were removed by centrifugation at 1000× *g* for 10 min at 4 °C. The resulting supernatant was subjected to centrifugation at 20,000× *g* for 30 min at 4 °C to obtain a membrane-enriched fraction, which was resuspended in Laemmli sample buffer. Protein concentration was determined using the Bradford assay. Equal amounts of protein (30 µg) were combined with Laemmli buffer containing 5% β-mercaptoethanol, denatured at 95 °C for 5 min, and resolved by SDS–polyacrylamide gel electrophoresis (12% acrylamide) under reducing conditions. Proteins were transferred onto nitrocellulose membranes using a wet electrotransfer system (100 V for 90 min at 4 °C). Membranes were blocked with 5% (*w*/*v*) non-fat dry milk in TBS-T (20 mM Tris-HCl, 150 mM NaCl, 0.1% Tween-20) for 1 h at room temperature and incubated overnight at 4 °C with a commercial mouse polyclonal anti-gp63 leishmanolysin-like antibody (USBiological Life Sciences, catalog no. 248216), generated against a conserved sequence within the gp63/leishmanolysin family of metalloproteases, diluted 1:1000 in blocking buffer. After washing, membranes were incubated with HRP-conjugated anti-mouse IgG secondary antibody (1:5000) for 1 h at room temperature. Immunoreactive bands were detected using enhanced chemiluminescence and documented with a digital imaging system.

### 2.10. Encystment Assays and Enrichment of Encysted Forms

Trophozoite cultures were initiated using an inoculum of 1 × 10^6^ cells in 25 cm^2^ culture plates containing Chang medium. Once 90–95% confluence was achieved, encystment analyses were initiated and maintained further for 7 days in the presence or absence of NSAIDs (100 µM) to evaluate the effect of these drugs on cyst formation. Cultures progressively differentiated into encysted forms, which were then collected by centrifugation at 2000 rpm for 20 min at room temperature. The resulting pellets were processed for analysis: cells were fixed with 4% paraformaldehyde in PBS and stained with Calcofluor White to detect β-linked polysaccharides in the cyst wall. Samples were analyzed using a Carl Zeiss LSM 900 confocal microscope with a 3 × 3 tile scan. Untreated cultures served as controls and were defined as 100% encystment.

For the detection of the gp63-like protein, immunofluorescence assays were performed independently of NSAID treatment. Anti-gp63 antibody and/or rhodamine–phalloidin were used to visualize gp63-like protein and actin, respectively. Preparations were analyzed by confocal fluorescence microscopy (LSM 900). All experiments included at least three independent biological replicates.

### 2.11. Domain Architecture Analysis of Leishmanolysin (gp63-like Protein)

The leishmanolysin (gp63) protein sequence from *Leishmania major* (UGS80635.1) was retrieved from the NCBI database [23]. The corresponding gp63-like protein sequence from *Acanthamoeba castellanii* was obtained from the Transcriptome Shotgun Assembly GJZG01, using accession XP_004337275.1 as reference, where it is annotated as a putative leishmanolysin. Domain organization and functional annotation were analyzed using InterProScan v5.77-108.0 [24]. Signal peptide prediction and subcellular localization were assessed using SignalP v6.0 [25] and DeepLoc v2.1 [26], respectively. Conserved motifs, including metal-binding sites and glycosylation motifs, were inferred using ScanProsite v 2025_03 [27], while disulfide bond predictions were performed with Cyscon v2015_09 [28]. Domain architecture representations were generated using IBS v2.0 [29]. Additional sequence refinement and annotation were performed using Geneious v8.1.9 software (Dotmatics, Boston, MA, USA) [30]. In *Leishmania* species, the gp63 and leishmanolysin terms are used as synonymous; hereafter, we refer to the *A. castellanii* protein as a gp63-like protein to indicate its putative homology.

### 2.12. Three-Dimensional Modeling of Leishmanolysin (gp63-like Protein) and NSAID Ligands

Three-dimensional structural models of the complete and mature activated forms of the leishmanolysin (gp63-like protein) from *A. castellanii* and *L. major* were generated for comparative analysis. Structural predictions were performed using ColabFold v1.5.5 [31], which implements AlphaFold2 coupled with MMseqs2. The best-ranked relaxed model for each protein was selected based on a global pLDDT confidence score above 90, as reported in comparable studies [32]. Model quality was evaluated using MolProbity v4.5.2 [33] and ProSA-web [34], while residue-level accuracy was assessed with ResQ v2016 [35]. Structural consistency was further validated by comparison with independent models generated using the SWISS-MODEL platform [36]. Final protein models were edited and visualized using ChimeraX v1.11.1 [37]. Structural alignment was performed using the MatchMaker tool of ChimeraX, employing the crystal structure of *L. major* gp63 (PDB: 1ML1) as a template. This alignment enabled the incorporation of the Zn^2+^ metallic cofactor required for the catalytic activity of mature leishmanolysin proteins, following removal of the signal peptide and proregion. The molecular and crystallographic structures of aspirin (CCDC: 185472), diclofenac (CCDC: 128771), and ibuprofen (CCDC: 1184450) were retrieved from the PubChem database [38]. Control compounds included amentoflavone, benzoic acid, and L-ascorbic acid. Protonation states were adjusted to physiological pH (~7.2) using Avogadro v1.2.0 [39], and initial energy minimization was performed with the MMFF94 force field. To ensure identification of global minimum conformations, conformational searches were conducted using CREST v6.2 with the GFN2-xTB method [40]. The lowest-energy conformers were subsequently optimized at the ωB97X-D4/def2-TZVPP level of theory using ORCA v6.1 [41], applying RIJCOSX approximation, CPCM solvation (water), and tight convergence criteria. Frequency analyses confirmed the absence of imaginary modes. Optimized ligand geometries were validated against experimental crystal structures using the MolGC v1.0 algorithm [42].

### 2.13. Molecular Docking Between Leishmanolysin (gp63-like Protein) and NSAIDs

Molecular docking simulations were performed to evaluate the interaction between NSAIDs and the mature forms of leishmanolysin (gp63-like protein) from *A. castellanii* and *L. major*. Ligand structures were prepared using AutoDockTools v1.5.7 [43], where non-polar hydrogens were merged and Gasteiger charges assigned. Protein protonation states at physiological pH (~7.2) were calculated using PDB2PQR v3.7.1 with the PROPKA algorithm [44]. Non-polar hydrogens were merged, and Kollman charges were assigned to generate final receptor models. Docking simulations were conducted using AutoDock, treating the gp63-like proteins as rigid receptors and NSAIDs as flexible ligands. The docking grid was centered on the catalytic pocket previously described for leishmanolysins [45], with grid dimensions of 50 × 50 × 50 points and a spacing of 0.375 Å. Docking calculations employed the Lamarckian Genetic Algorithm with 150 independent runs, 25,000,000 energy evaluations, and 270,000 generations. Docking poses were ranked based on binding energy and clustering criteria. Protein–ligand interactions were visualized and analyzed using the Protein–Ligand Interaction Profiler v3.0 [46] and BIOVIA Discovery Studio Visualizer (Dassault Systèmes SE, Vélizy-Villacoublay, France) [47].

### 2.14. Toxicological and Pharmacological Predictions

The chemical structures and SMILES notations of ibuprofen, aspirin, and diclofenac were obtained from PubChem. Pharmacokinetic properties, bioavailability, and drug-likeness were predicted using SwissADMEv2019, SIB © [48]. Structural and pharmacophoric similarity analyses were conducted using SwissSimilarity (v2021, SIB©) [49,50], while potential human protein targets were predicted using SwissTargetPrediction (v2017, SIB©) [51]. These bioinformatic tools come from SwissDrugDesign © (Swiss Institute of Bioinformatics; Lausanne, Switzerland (https://www.molecular-modelling.ch/swiss-drug-design.html (accessed on 1 August 2025)).

Additional toxicological and ADMET predictions were performed using the CompTox Chemicals Dashboard (v2.5.1, EPA; Durham, NC, USA) (https://comptox.epa.gov/dashboard/ (accessed on 1 August 2025)) [52], the Similarity Ensemble Approach (v1.0, UCSF; San Francisco, CA, USA) (https://sea.bkslab.org/ (accessed on 1 August 2025)) [53], and admetSAR (v3.0, East China University of Science and Technology; Shangai, China) (https://lmmd.ecust.edu.cn/admetsar3/index.php (accessed on 1 August 2025)) [54,55]. Finally, IC_50_ values for the compounds in human cancer and non-tumorigenic cell lines were retrieved from the ChEMBL database (EMBL-EBI Genome Campus; Hinxton, Cambridgeshire, UK) (https://www.ebi.ac.uk/chembl/ (accessed on 1 August 2025)) [56].

## 3. Results

### 3.1. Non-Steroidal Anti-Inflammatory Drugs (NSAIDs) Do Not Affect the Growth of A. castellanii Trophozoites but Selectively Impair Adhesion

Adhesion of *Acanthamoeba castellanii* trophozoites to abiotic surfaces or host tissues represents a critical early event in colonization and pathogenicity and depends on coordinated surface interactions and cytoskeletal dynamics. Given the reported involvement of cyclooxygenase-related pathways in protozoan differentiation and host interaction, we evaluated the effects of aspirin, ibuprofen, and diclofenac on trophozoite growth, viability, and adhesion.

**Figure 1:**
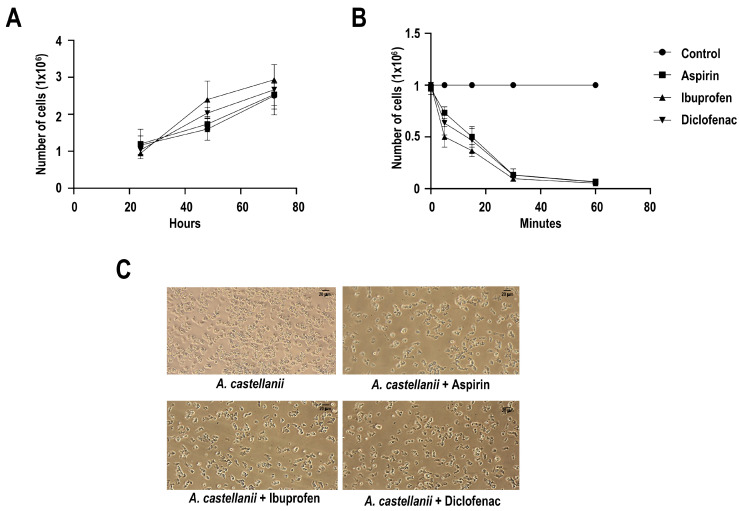
**Effects of NSAIDs on growth, viability, and adhesion of** ***Acanthamoeba castellanii*** **trophozoites.** (**A**) Trophozoites (2.5 × 10^5^) were exposed to NSAIDs (aspirin, ibuprofen, or diclofenac; 100 μM) for 24, 48, and 72 h in Chang’s medium. Total, adherent, and non-adherent cells were quantified using a Neubauer chamber. Viability was assessed by trypan blue exclusion. (**B**) Adhesion assays were performed using trophozoites precultured to 95% confluence (1 × 10^6^ cells) and treated with 100 μM of each NSAID. Adhesion was evaluated at 5, 15, 30, and 60 min. Non-adherent cells were removed by decanting and washing with Chang medium. Adherent cells were subsequently recovered by incubation on ice, centrifugation, and resuspension in PBS prior to cell counting. (**C**) Representative optical microscopy images of trophozoite cultures under the indicated experimental conditions. All assays were performed in triplicate in three independent experiments.

Treatment with NSAIDs did not significantly affect trophozoite proliferation over a 72 h period, nor did it compromise parasite viability, as assessed by trypan blue exclusion (Figure S1). This analysis revealed no significant effect of drug treatment on parasite growth compared with untreated controls (Figure 1A). In contrast, exposure to aspirin, ibuprofen, or diclofenac resulted in a rapid and marked reduction in trophozoite adhesion. This analysis demonstrated a significant effect of NSAIDs treatment on adhesion dynamics over time compared with controls (Figure 1B). Representative phase-contrast micrographs illustrating trophozoite morphology and attachment under each experimental condition are shown in Figure 1C. Collectively, these results indicate that NSAIDs selectively disrupt early adhesion processes in *A. castellanii* without impairing trophozoite growth or survival. All assays were performed in triplicate across three independent biological replicates.

### 3.2. Brain Tissue Discontinuous Micropatterns (BTDM) Enhance A. castellanii Motility and gp63/Leishmanolysin-like Protein–Actin Association

**Figure 2:**
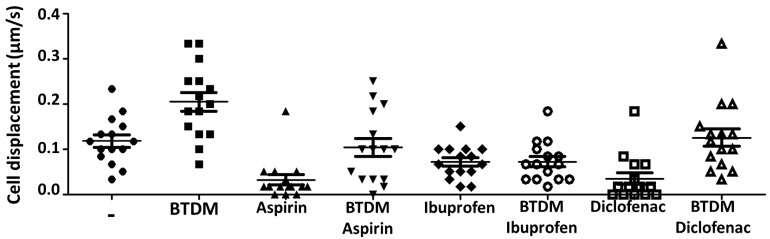
***Acanthamoeba castellanii*** **trophozoites displacement after stimulation with BTDM and treatment with NSAIDs.** Trophozoite migration was monitored by inverted light microscopy (Nikon Diaphot, 20× objective). After stimulation with BTDM for 1 h, cell displacement was recorded for 60 s, using the optical field diameter (1.940 mm) as a reference. Trophozoites were subsequently incubated with NSAIDs, including aspirin, ibuprofen, and diclofenac (100 μM each). Migration speed was calculated and expressed as µm/s. Each symbol represents an individual trophozoite (*n* = 15 per condition), and horizontal lines indicate mean ± SEM. Data correspond to three independent biological replicates. Statistical analysis was performed using one-way ANOVA followed by Bonferroni’s multiple comparison test.

**Figure 3:**
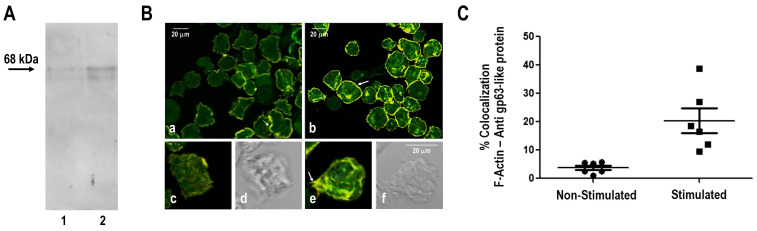
Expression of gp63 and organization of the actin cytoskeleton in *Acanthamoeba castellanii* incubated with discontinuous micropatterns of mouse brain tissue (BTDM). (**A**) Western blot analysis of gp63-like protein (Leishmanolysin) expression in trophozoites. Lane 1 corresponds to membrane fractions from trophozoites incubated without BTDM, and lane 2 corresponds to trophozoites incubated with BTDM for 1 h. gp63 was detected using a commercial anti-gp63 antibody. (**B**) Confocal fluorescence microscopy images showing F-actin (rhodamine–phalloidin, red) and gp63-like protein (green). Co-localization appears in yellow. (**a**–**e**). Trophozoites cultured without BTDM (**a**,**c**) and trophozoites cultured with BTDM for 1 h (**b**,**e**). Higher magnification of individual cells highlighting co-localization ((**c**) = without BTDM; (**e**) = with BTDM). In (**d**,**f**), corresponds to DIC analysis. Arrows indicate representative areas of co-localization. Scale bars = 20 μm. (**C**) Quantitative analysis of the colocalization between filamentous actin and the gp63-like protein, based on fluorescence signal intensity, in trophozoites cultured with or without BTDM stimulation. Non-stimulated trophozoites exhibited a mean F-actin/gp63-like protein colocalization of approximately 3.8, whereas BTDM-stimulated trophozoites showed an increase to approximately 20% (*n* = 6 trophozoites). Total fluorescence intensity and colocalization between the red (F-actin) and green (gp63-like protein) signals were quantified using ImageJ 2 software. Data represent three independent biological replicates (*n* = 3) and are expressed as mean ± SEM.

Trophozoite adhesion is essential for migration and invasion, requiring both substrate anchoring and cytoskeletal reorganization. To examine the involvement of cyclooxygenase-sensitive pathways, we evaluated NSAIDs effects on *A. castellanii* motility and gp63-like protein distribution using BTDM, a biologically relevant substrate derived from an organ preferentially invaded by the parasite. Under control conditions, BTDM stimulation significantly increased mean trophozoite displacement velocity to 0.205 µm/s compared with 0.117 µm/s in unstimulated conditions (paired *t*-test, *n* = 6, *p* = 0.003) (Figure 2). NSAIDs treatment markedly impaired adhesion, leaving a fraction of non-adherent trophozoites. Among attached cells, ibuprofen abolished the motility increase induced by BTDM, whereas aspirin and diclofenac partially reduced motility. Immunoblot assays confirmed the presence of a protein recognized by the monoclonal anti gp63 antibody against *Leishmania mexicana* gp63 (Figure 3A); confocal analyses further confirmed that the gp63-like protein (~68 kDa) in *A. castellanii* showed a spatial association with filamentous actin. BTDM stimulation enhanced this spatial association of gp63-like protein with filamentous actin, particularly within acanthopodial projections involved in substrate interaction and motility (Figure 3B(b,e)). In unstimulated trophozoites, gp63-actin association was lower (Figure 3B(a,c)). This result suggested that the gp63-like protein localized predominantly at the amoeba surface, and BTDM stimulation enhanced its association with F-actin structures (Figure 3C), particularly within acanthopodial projections (Figure 3B(e)). These observations indicate that NSAIDs modulate both host cytoskeletal responses and amoeba gp63-actin dynamics during host–pathogen interactions.

### 3.3. NSAIDs Affect Actin Filament Organization in MDCK Cells During A. castellanii–MDCK Cell Interactions

**Figure 4:**
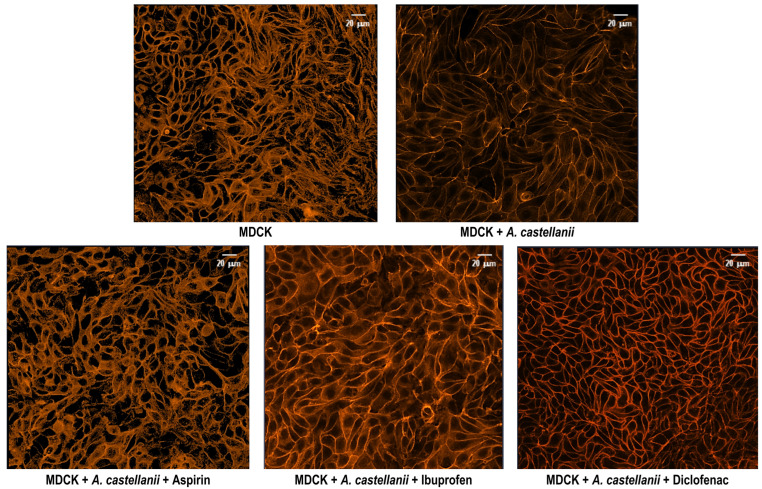
**Actin cytoskeleton organization in MDCK cells exposed to** ***Acanthamoeba castellanii*** **trophozoites and NSAIDs**. MDCK cell monolayers were stained with rhodamine–phalloidin to visualize filamentous actin (F-actin). Confocal microscopy images were acquired using a Carl Zeiss LSM 900 confocal microscope equipped with a 40× oil-immersion objective (numerical aperture, NA 1.3), showing rhodamine–phalloidin-labeled F-actin in MDCK cells incubated in the presence of *A. castellanii* trophozoites and the indicated drugs. A control condition without trophozoites was included to assess basal actin organization. Representative tile-scan images (3 × 3 fields) obtained at 40× magnification are shown, illustrating multiple regions of each culture to increase field coverage and minimize potential bias associated with single-field selection.

To determine whether substrate-mediated effects were reproduced in a more physiologically relevant context, we examined F-actin organization in MDCK cells co-cultured with *A. castellanii* trophozoites. F-actin was visualized using rhodamine-phalloidin staining, and confocal microscopy was employed to assess cytoskeletal rearrangements in the presence or absence of NSAIDs. Under control conditions, MDCK cells exhibited a well-organized actin cytoskeleton, with stress fibers, cortical actin beneath the plasma membrane, and a fine cytoplasmic network. Co-culture with *A. castellanii* resulted in cortical actin predominance, loss of stress fibers, and reduced polymerized actin. NSAID treatment differentially modulated these changes. Aspirin-treated MDCK cells retained visible stress fibers, whereas diclofenac- or ibuprofen-treated cells displayed actin patterns comparable to untreated co-cultures, with cortical actin predominance and reduced polymerized actin (Figure S2). Quantitative fluorescence intensity analysis further showed that co-incubation with trophozoites decreased the F-actin signal to approximately 50% relative to untreated MDCK controls, whereas NSAID-treated co-cultures preserved fluorescence intensity without major alterations (Figure S3). To minimize potential single-field bias, a tile scan composed of nine adjacent fields was analyzed. This approach suggest that both the NSAID-dependent actin reorganization and the reduction in fluorescence intensity represented general effects across the MDCK cell monolayer during interaction with *A. castellanii*. Figure 4 shows representative confocal micrographs from each condition, obtained from three independent biological replicates.

### 3.4. Cyclooxygenase Inhibitors Differentially Modulate Encystment of A. castellanii

**Figure 5:**
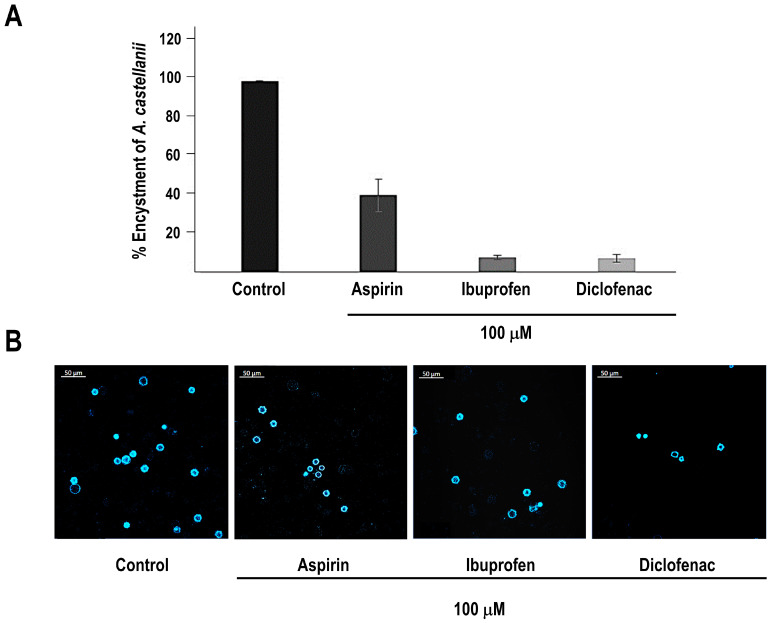
**NSAIDs differentially inhibit encystment in** ***Acanthamoeba castellanii*****.** (**A**) Quantitative analysis of cyst formation in untreated controls and in cells treated with aspirin, diclofenac, or ibuprofen. Data are expressed as the percentage of cysts relative to the untreated control and represent the mean ± SD of three independent biological replicates. (**B**) Fluorescence micrographs corresponding to each experimental condition, illustrating the reduction in cyst number upon NSAID treatment. Scale bar: 50 µm.

To determine whether cyclooxygenase-sensitive pathways participate in cyst differentiation, we evaluated the effects of NSAIDs with distinct pharmacological profiles on *A. castellanii* encystment. Quantitative analysis revealed that aspirin treatment reduced the formation of cysts by approximately 60% compared with untreated controls. In contrast, diclofenac and ibuprofen produced an inhibitory effect, reducing cyst formation by nearly 90%. Data represent the mean ± SD of three independent biological replicates (Figure 5A). Representative fluorescence micrographs corresponding to each condition are shown in the lower panel of Figure 5B. Collectively, these results indicate that encystment is differentially sensitive to NSAID treatment, suggesting the involvement of cyclooxygenase-like or NSAID-responsive pathways during cyst differentiation.

### 3.5. Distribution of gp63-like Protein in Cyst Wall Structures During Encystment

**Figure 6:**
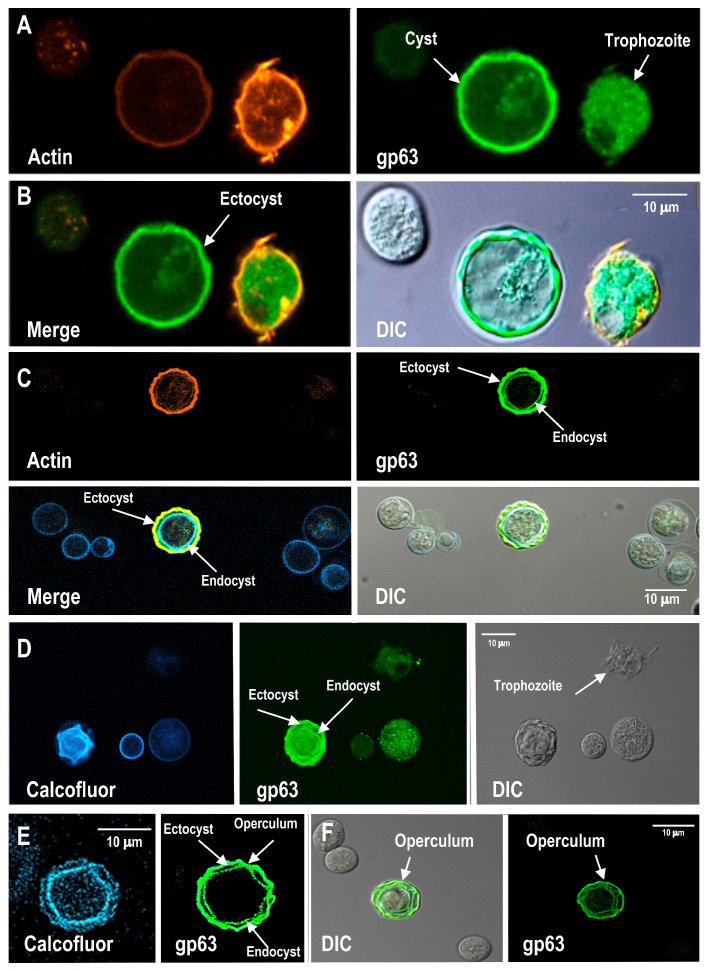
Confocal immunofluorescence microscopy analysis of gp63-like protein and actin distribution in trophozoites and cysts. (**A**) Representative confocal images showing actin staining (orange) and gp63-like protein labeling (green) in trophozoites and cysts. Actin displayed a more intense signal in trophozoites, whereas gp63 was detected in both stages. (**B**) Merged images illustrating partial colocalization of gp63 and actin, predominantly at the trophozoite plasmatic membrane. In the case of ectocyst, the presence of gp63-like protein is abundant (arrow). Differential interference contrast (DIC) images confirm cyst and trophozoite morphology. (**C**) Confocal images of mature cysts showing gp63-like protein localization in both the ectocyst and endocyst layers (arrows). Actin signal was more abundant in the ectocyst, as observed in merged images. DIC images highlight the differentiation of cyst layers. (**D**) Calcofluor staining (blue), analyzed by confocal microscopy, uniformly cyst walls. gp63-like protein labeling (green) was detected in cysts with well-defined ectocyst and endocyst layers, while DIC imaging revealed a subpopulation of calcofluor-positive cysts lacking detectable gp63-like protein signal. (**E**,**F**) Confocal gp63 localization at the operculum, as evidenced by immunofluorescence and confirmed by DIC imaging (arrows). (**E**) Higher-magnification confocal images show gp63-like protein localization in the ectocyst, endocyst, and operculum (arrows). Scale bars = 10 μm.

We decided to investigate the subcellular localization of the gp63-like protein and actin during trophozoite-to-cyst differentiation. Enriched *A. castellanii* cysts were stained with Calcofluor White to visualize β-linked polysaccharides in the cyst wall (Figure S4a). Differential interference contrast (DIC) microscopy was used to examine cyst morphology, as shown in Figure S4b. Enriched encysted-form preparations, obtained after 7 days of differentiation, were analyzed. No SDS or other selective purification procedures were applied; therefore, preparations may have contained cysts at different maturation stages. Immunofluorescence analysis was performed to examine the spatial distribution of the gp63-like protein relative to actin, allowing assessment of possible associations that could contribute to the differential effects of NSAIDs on encystation. Immunofluorescence analysis using a commercial anti-*Leishmania major* gp63 antibody revealed distinct localization patterns of gp63-like protein and actin in trophozoites and cysts (Figure 6A, upper panels). In trophozoites, actin displayed an intense and relatively homogeneous distribution, supporting its role in substrate adhesion, whereas in cysts the actin signal was reduced and spatially restricted (Figure 6A, left panel). gp63-like immunoreactivity was detected in both forms, with prominent peripheral localization in cysts. Merged images revealed partial colocalization of gp63-like protein and actin, predominantly at the cyst periphery corresponding to the ectocyst, suggesting that gp63-like may interact with actin-based structures to facilitate cytoskeletal remodeling and cyst wall formation (Figure 6B, left panel).

Within the enriched cyst preparations, morphologically mature forms were occasionally identifiable. In these cysts, the gp63-like protein appeared to localize to both ectocyst and endocyst layers, as well as to the opercular region (Figure 6C–F). In one illustrative example (Figure 6C, lower left), actin and gp63-like protein signals showed partial colocalization (Pearson’s correlation coefficient of approximately 40%), suggesting a moderate spatial association between gp63-like protein and actin. Notably, a subset of Calcofluor-positive cysts lacked detectable gp63-like immunoreactivity, indicating variability in expression or localization during cyst maturation [4].

These observations provide descriptive insights into the distribution of gp63-like protein and actin. Although the analyzed population included cysts at various developmental stages, focusing on morphologically mature forms allowed us to capture general patterns of protein localization without implying definitive mechanistic interactions. The precise subcellular localization of gp63-like protein in *A. castellanii* cysts remains to be determined and could be further clarified using high-resolution approaches, such as transmission electron microscopy with gold-labeled antibodies. Future studies using synchronized cyst populations will be required to evaluate stage-specific localization and potential functional roles of gp63-like protein in fully mature cysts [4].

### 3.6. Structural Comparison of gp63-like Proteins from A. castellanii and L. major

**Figure 7:**
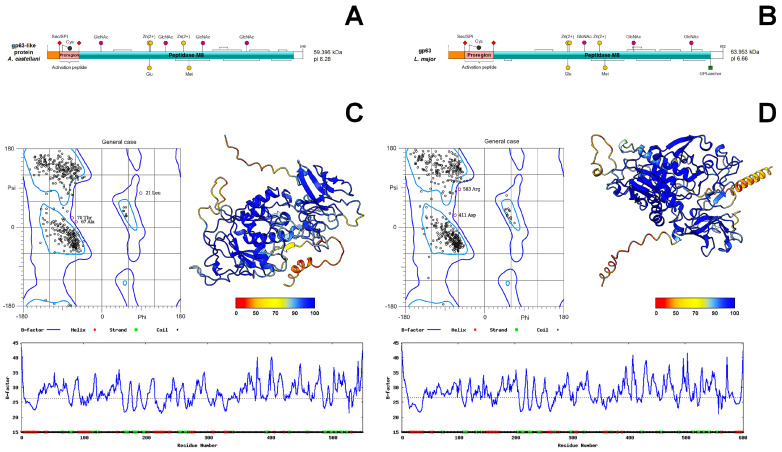
**Comparative domain architecture and structural analysis of gp63 metalloproteases from *Acanthamoeba castellanii* and *Leishmania major*.** (**A**,**C**) gp63 from *A. castellanii*; (**B**,**D**) gp63 from *L. major*. (**A**,**B**) Schematic representation of gp63 domain organization showing predicted signal peptide (orange), activating proregion (pink), and catalytic peptidase M8 domain (aquamarine); sites of glycosylation (GlcNAc), metal-binding (Zn^2+^), and other post-translational modifications are also pointed out. The theoretical molecular weight (kDa) and isoelectric point (pI) are indicated for each protein. (**C**,**D**) Structural validation of the predicted 3D folding models. (**Upper left**) Ramachandran plots with most residues in favored regions and few outliers (pink circles). (**Upper right**) 3D ribbon models of gp63 colored by confidence score (pLDDT). (**Bottom**) B-factor plots illustrating residue flexibility and secondary structure assignment.

A putative leishmanolysin sequence was identified in the *A. castellanii* transcriptome and compared with the *L. major* gp63. Domain architecture analysis revealed a conserved organization characteristic of M8 family metalloproteases, including an N-terminal signal peptide (Figure S5B,C), a cleavable inhibitory proregion, and a C-terminal catalytic domain (Figure 7A,B). The catalytic region contains conserved disulfide bridges, N-glycosylation sites, and zinc-coordinating residues essential for enzymatic activity in the active site by the HEXXH(AL)G(FS) motif (Figure S5A).

Despite low overall sequence identity (24.6%), the *A. castellanii* gp63-like protein and leishmanolysin of *L. major* share a conserved catalytic architecture (Figure S5A). A notable difference is the presence of a C-terminal GPI-anchoring motif in *L. major* protein, which is absent in the *A. castellanii* gp63-like, suggesting a non-GPI-anchored or potentially secreted form (Figure 7A,B).

Three-dimensional modeling revealed conserved folds typical of zinc-dependent metalloproteases, with high confidence scores in catalytic regions and greater flexibility in terminal and loop regions (Figure 7C,D). These analyses suggest conservation of catalytic architecture despite species-specific structural differences.

### 3.7. Molecular Docking Analysis of gp63–NSAID Interactions

**Figure 8:**
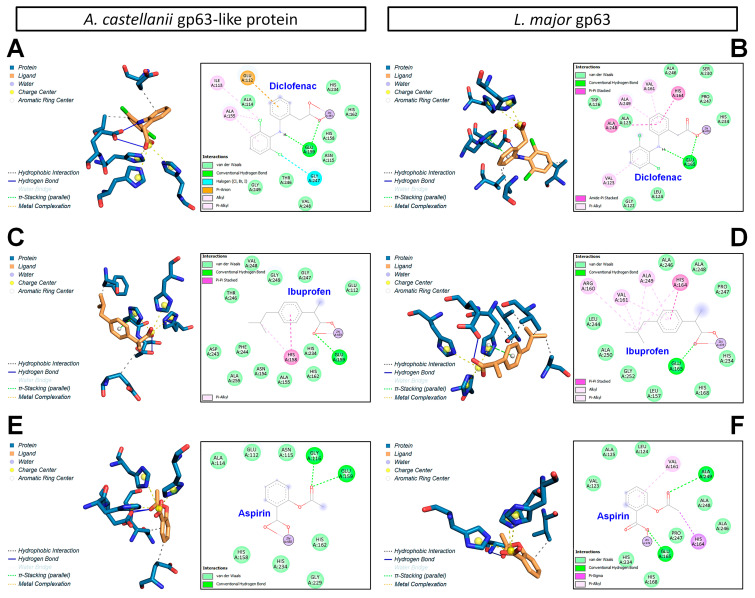
Predicted binding interactions of nonsteroidal anti-inflammatory drugs (NSAIDs) with gp63 metalloproteases from *A. castellanii* and *L. major*. (**A**,**C**,**E**) gp63 of *A. castellanii*; (**B**,**D**,**F**) gp63 of *L. major*. Docking interactions of diclofenac (**A**,**B**); ibuprofen (**C**,**D**); aspirin (**E**,**F**). Protein residues are shown in blue and ligands in orange. Yellow dashed lines highlight metal-complex interactions involving Zn^2+^; and gray dashed lines denote hydrophobic contacts. Residues involved in ligand stabilization are detailed in 2D maps (black squares). Two-dimensional interaction diagrams for diclofenac, ibuprofen, and aspirin, showing the specific amino acid residues involved in ligand binding. The interactions include van der Waals forces, conventional hydrogen bonds in green dashed lines, and coordination with the catalytic metal ion in red lines.

Docking analyses showed that aspirin, ibuprofen, and diclofenac interact with the catalytic pocket of gp63 from both *A. castellanii* and *L. major*, including coordination with the catalytic zinc ion stabilized by conserved histidine residues (Figure 8). Binding was dominated by hydrophobic interactions, with limited hydrogen bonding involving a conserved glutamate residue. Diclofenac exhibited the strongest predicted binding affinity, followed by ibuprofen and aspirin (Table 1). Interaction patterns were largely conserved between species, with minor variations reflecting differences in pocket geometry.

### 3.8. In Silico Assessment of Toxicity and Pharmacological Profiles of NSAIDs

To assess the drug-likeness and safety profiles of ibuprofen, aspirin, and diclofenac in the context of their potential repurposing against *Acanthamoeba castellanii*, comprehensive in silico cheminformatics analyses of ADME properties and toxicity were conducted. All three NSAIDs complied with Lipinski’s rule-of-five, supporting their suitability as orally bioavailable small-molecule candidates (Figure S6). Quantitative estimation of drug-likeness (QED) revealed compound-dependent variability, with diclofenac showing the highest score (96.5%), followed by ibuprofen (89.5%) and aspirin (57.1%) (Table 2). Notably, aspirin was the only compound predicted to satisfy both Pfizer and GlaxoSmithKline safety model criteria (Figure S6B).

Predicted absorption and distribution parameters indicated moderate intestinal absorption, membrane permeability, and aqueous solubility for all compounds (Table 2). Passive diffusion across the blood–brain barrier was predicted for each NSAID, and none were identified as substrates of P-glycoprotein (Figure S6). ADME profiling further suggested predominant renal clearance and phase II metabolism, mainly via UGT-mediated glucuronidation (Figure S6A–C).

Regarding metabolism- and safety-related liabilities, ibuprofen and diclofenac were predicted to inhibit cytochrome P450 enzymes. Diclofenac additionally exhibited predicted inhibition of the bile salt export pump, as well as potential interactions with peroxisome proliferator-activated receptor gamma (PPARγ) and matrix metalloproteinases (Figure S6C). Cytotoxicity predictions revealed compound-specific profiles, with ibuprofen displaying the lowest predicted cytotoxicity (IC_50_ ≈ 983.75 μM), followed by aspirin (IC_50_ ≈ 727.12 μM), whereas diclofenac showed the highest predicted toxicity (IC_50_ ≈ 135.14 μM) (Table 2; Figure S6A–C). Both ibuprofen and diclofenac were predicted to inhibit human ether-à-go-go-related gene (hERG) channels at concentrations of approximately 30 μM (Figure S6A,C).

Target prediction analysis indicated that all three NSAIDs predominantly interact with oxidoreductases, class A G protein-coupled receptors, and enzyme families. Ibuprofen and aspirin additionally showed predicted interactions with electrochemical transporters, whereas diclofenac exhibited a broader target spectrum, including proteins associated with secretion pathways (Figure S6A–C). Structural and pharmacophore similarity analyses identified partial similarity between ibuprofen and propachlor (23%), aspirin and diethyl phthalate (30%), and diclofenac and 2,6-dichlorobenzamide (25%) (Figure S7) (Table 2), highlighting potential host-related safety considerations associated with these chemical scaffolds.

## 4. Discussion

Treatment of trophozoites with aspirin, ibuprofen, or diclofenac selectively impaired adhesion and migration, whereas parasite proliferation and viability remained largely unaffected under the experimental conditions tested. This selective response supports the interpretation that NSAIDs interfere with specific regulatory or signaling pathways rather than inducing generalized cytotoxicity. Because adhesion and motility represent early and essential steps in *A. castellanii* colonization and host–parasite interaction, disruption of these processes is likely to influence pathogenic potential. These findings should therefore be interpreted within an exploratory drug repurposing framework. The use of commercial pharmaceutical formulations constitutes a methodological limitation, as excipients may contribute to the observed effects. Accordingly, the results are hypothesis-generating rather than definitive mechanistic evidence.

The differential inhibition of trophozoite adhesion among aspirin, diclofenac, and ibuprofen may relate to their distinct effects on cytoskeletal dynamics. Adhesion and migration in *A. castellanii* depend on actin-mediated rearrangements required for acanthopod formation and substrate attachment [57]. Diclofenac and ibuprofen have been reported to interfere with actin polymerization and cytoskeletal organization in eukaryotic cells, altering cell shape, motility, and adhesion. These effects have been linked to modulation of Rho GTPase-dependent signaling and actin stress fiber reorganization, including in models treated with ibuprofen, frequently accompanied by changes in calcium signaling and actin-binding proteins involved in amoeboid movement [58].

In contrast, aspirin primarily acts through irreversible acetylation of cyclooxygenase enzymes. Alterations in actin organization following aspirin exposure have been described in *Entamoeba histolytica* [59]. In the present study, aspirin exerted a comparatively weaker inhibitory effect on adhesion than diclofenac and ibuprofen. These observations are descriptive and do not imply a defined molecular mechanism.

Although NSAIDs may affect actin-related signaling, their potential impact on cyclooxygenase-like activity in *A. castellanii* cannot be excluded. Molecular docking analyses indicated structural plausibility of NSAID association with conserved regions within the catalytic pocket of a gp63-like protein. However, these computational predictions are hypothesis-generating and do not demonstrate biological binding or enzymatic inhibition. The gp63-like protein should therefore be regarded as a putative molecular component rather than a confirmed target, pending biochemical and genetic validation.

These findings are consistent with both reports describing modulation of cyclooxygenase-related pathways during experimental Acanthamoeba infections [60] and in vitro studies suggesting that NSAID-mediated inhibition of cyclooxygenase activity may influence encystment [19]. However, unlike Siddiqui et al. (2016) [19], who reported growth inhibition following NSAID exposure, no detectable effect on proliferation was observed here. Methodological differences may explain this discrepancy. Siddiqui et al. used diclofenac tablets dissolved in ethanol, whereas the present study employed an injectable diclofenac formulation lacking ethanol and prepared directly in Chang medium at 100 µM. Differences in solvent systems and excipient composition may influence cellular responses and should be considered when comparing studies.

Using BTDM as a biologically relevant substrate, enhanced trophozoite motility was observed together with increased surface localization and actin-associated distribution of the gp63-like protein. These findings align with previous studies suggesting that substrate and microenvironmental cues influence actin organization and surface protein localization in *A. castellanii* [1,57,61]. While these observations provide descriptive insight into parasite–substrate interactions, they do not establish a mechanistic link. Further investigation is required to clarify the relationship between substrate cues, protein localization, and cytoskeletal dynamics.

A gp63-like protein has been identified in *A. castellanii* sharing conserved structural features with leishmanial gp63, including motifs characteristic of zinc-dependent metalloproteases of the M8 family [15]. Detection was achieved using the D12 monoclonal antibody [14]; therefore, the designation “gp63-like protein” reflects structural similarity rather than confirmed enzymatic identity.

In other protozoan parasites, particularly Leishmania, gp63 facilitates adhesion, complement receptor interaction, and host cell engagement, contributing to virulence and cytoskeletal modulation [62,63]. The cortical association observed in motile trophozoites suggests possible functional analogy, although direct biochemical evidence in *A. castellanii* remains to be demonstrated.

Encystment assays revealed marked sensitivity to NSAID exposure, with diclofenac producing the strongest reduction in cyst formation. This suggests that NSAID-responsive pathways may intersect with developmental transitions in *A. castellanii*. Immunofluorescence analyses demonstrated that gp63-like protein is associated not only with trophozoites but also with cyst wall-related structures, including ectocyst, endocyst, and opercular regions. Calcofluor White staining confirmed the presence of polysaccharide-rich cyst walls structures and also confirmed the presence of encysted forms within a cyst-enriched population; differential interference contrast microscopy documented also morphological integrity.

Partial spatial overlap between gp63-like protein and actin was detected predominantly at the cyst periphery. Quantitative colocalization analysis based on fluorescence intensity measurements and Pearson’s coefficient yielded an average overlap of approximately 40%, indicating moderate spatial association. These observations are consistent with prior reports describing dynamic reorganization of filamentous actin during encystment and cyst wall assembly. Notably, a subset of Calcofluor-positive encysted forms lacked detectable gp63-like immunoreactivity, suggesting variability in gp63-like protein localization or expression across developmental stages. Because cyst-enriched populations were analyzed without detergent-based purification of fully mature cysts [4], the observed heterogeneity likely reflects temporal variation during cyst wall formation rather than experimental inconsistency. The aim was not to define gp63-like protein as a fixed structural component of mature cyst walls but to document its association with encystment as a dynamic process.

Structural modeling indicated that the gp63-like protein shares key architectural features with M8 family metalloproteinases such as leishmanolysin from *Leishmania major* and *Schistosoma japonicum* [64]. These include a catalytic core composed of parallel β-sheets flanked by α-helices and the conserved HEXXH(AL)G(FS) motif involved in zinc coordination [64,65]. Docking analyses predicted that aspirin, ibuprofen, and diclofenac may associate within regions of the putative catalytic pocket, particularly near residues involved in zinc binding and substrate recognition.

Predicted interactions were primarily driven by hydrophobic complementarity with selective polar stabilization, similar to binding modes described for non-canonical ligands interacting with leishmanolysins from *Leishmania panamensis* and *L. mexicana* [15,45]. However, these findings represent structural compatibility rather than evidence of enzymatic inhibition.

Although the present study does not demonstrate proteolytic activity attributable to gp63-like protein, the phenotypic effects observed—particularly reduced adhesion and migration—are comparable to those reported in other parasitic systems involving gp63-related proteins [64]. The drug concentrations employed did not reach broadly cytotoxic levels, reducing but not excluding the possibility of nonspecific effects. Therefore, functional assignment of this protein remains provisional and requires targeted biochemical, proteomic, and genetic validation.

In silico pharmacokinetic and toxicological analyses were incorporated to contextualize the findings within a broader pharmacological framework [66,67]. The physicochemical profiles of aspirin, ibuprofen, and diclofenac are consistent with efficient absorption, passive diffusion, and intracellular accessibility. Predicted blood–brain barrier penetration aligns with known CNS distribution of several NSAIDs [68,69,70], which is relevant given the neurotropic potential of Acanthamoeba. ADME predictions suggested predominant phase II metabolism via glucuronidation and compound-specific interactions with cytochrome P450 enzymes [71,72], highlighting differences in host safety profiles. Ibuprofen displayed a comparatively favorable predicted tolerance profile, supporting its use as a probe compound for mechanistic exploration, whereas diclofenac’s broader interaction spectrum underscores the need for cautious interpretation due to potential pleiotropic effects.

Target prediction and structural similarity analyses emphasize the non-selective nature of NSAIDs and their capacity to engage diverse enzyme families and signaling pathways [73]. This pharmacological breadth may explain why NSAID exposure selectively affected adhesion, motility, and encystment without markedly impairing proliferation. Importantly, these computational predictions are hypothesis-generating and require direct experimental validation to determine whether modulation of NSAID-responsive pathways produces biologically meaningful effects on *A. castellanii* pathogenic traits [74,75,76].

## 5. Conclusions

In summary, non-steroidal anti-inflammatory drugs selectively interfere with key pathogenic characteristics of *Acanthamoeba castellanii*, including adhesion, motility, and encystment. These effects lead to the proposed involvement of NSAID-sensitive pathways in processes central to amoebic persistence and host interaction. While the data support a contextual association with a gp63-like protein structural framework, they do not establish a defined molecular mechanism. This work provides a conceptual foundation for future studies aimed at dissecting the molecular basis and biological relevance of these NSAID-sensitive pathogenic processes.

## Figures and Tables

**Table 1:** Predicted binding affinities derived from molecular docking of NSAIDs with *A. castellanii* gp63-like protein.

Compound	Sample Type	Binding Energy (kcal·mol^−1^)	Inhibition Constant (Ki, 25 °C)
L-Ascorbic acid	Low affinity or low specificity (control)	−4.31	689.74 µM
Benzoic acid	Moderate affinity (control)	−7.2	5.27 µM
Aspirin	Sample	−7.85	1.76 µM
Ibuprofen	Sample	−9.49	0.111 µM
Diclofenac	Sample	−10.37	0.025 µM
Amentoflavone	High affinity (control)	−10.73	0.014 µM

Binding energies and inhibition constants were obtained from AutoDock calculations at 298.15 K. Root-mean-square deviation (RMSD) tolerance = 2.0 Å.

**Table 2:** In silico pharmacological and toxicity profile of NSAIDs evaluated against *A. castellanii*.

Parameter	Ibuprofen	Aspirin	Diclofenac
Lipinski criteria (%)	100	100	100
QED score (%)	89.5	57.1	96.5
Permeability (%)	13.64	23.26	18.04
Solubility (%)	51.48	34.9	63.63
IC_50_ (theoretical) (μM)	≈983.75 ± 424	≈727.12 ± 137	≈135.14 ± 56
AC_50_ (theoretical) (μM)	144	186	-
BMD (μM)	50	90	90
CSTA (%)	Propachlor (23)	Diethyl phthalate (30)	Dichlorobenzamide (25)
Main molecular target	Oxidoreductases, transporters	Prostaglandin synthases	Prostaglandins, chemokine
Predicted toxicity	Low	Moderate	High

QED, quantitative estimation of drug-likeness; IC_50_, inhibitory concentration 50%; AC_50_, activating concentration 50%; BMD, benchmark dose; CSTA, chemical similarity of toxic analogs.

## Data Availability

The original contributions presented in this study are included in the article/Supplementary Materials. Further inquiries can be directed to the corresponding author.

## Supplementary Materials

The following supporting information can be downloaded at: https://www.mdpi.com/article/10.3390/pathogens15030263/s1, Figure S1: Cell viability assay of *Acanthamoeba castellanii* trophozoites during adhesion kinetics. Figure S2: Actin cytoskeleton organization in MDCK cells co-cultured with *Acanthamoeba castellanii* trophozoites and NSAIDs. Figure S3: Confocal analysis of *Acanthamoeba castellanii* cysts. Figure S4: Protein sequence alignment and predicted subcellular localization signals of gp63 metalloproteases. Figure S5: In silico ADME, drug-likeness, and toxicity prediction profiles of NSAIDs evaluated against *Acanthamoeba castellanii*. Figure S6: In silico ADME, drug-likeness, and toxicity prediction profiles of NSAIDs evaluated against *Acanthamoeba castellanii*. Figure S7. Structural similarity profiles of NSAIDs and selected toxic analogs. Table S1: Preparation of NSAID stock solutions, working concentrations, and vehicle controls.

## Abbreviations

The following abbreviations are used in this manuscript:

AC_50_	Activating concentration 50%
BMD	Benchmark dose
BTDM	Brain tissue discontinuous micropatterns
COX	Cyclooxygenase
CSTA	Chemical similarity of toxic analogs
DAPI	4′,6-diamidino-2-fenilindol
DIC	Differential interference contrast
DMEM	Dulbecco’s modified Eagle medium
DMSO	Dimethyl sulfoxide
FBS	Fetal bovine serum
hERG	Human ether-à-go-go-related gene
IC_50_	Inhibitory concentration 50%
Ki	Inhibition constant
MDCK	Madin–Darby canine kidney
NSAIDs	Non-steroidal anti-inflammatory drugs
pI	Isoelectric point
PPARγ	Peroxisome proliferator-activated receptor gamma
QED	Quantitative estimation of drug-likeness
RMSD	Root-mean-square deviation
