# Supplementary material for: Acanthamoeba castellanii: Non-Steroidal Anti-Inflammatory Drugs Affect Adhesion, Motility, and Encystment, Suggesting a Link with a gp63-like Protein Candidate
Source: Pathogens. 2026 Mar 2;15(3):263. doi: 10.3390/pathogens15030263 (PMC13029602; doi:10.3390/pathogens15030263)
Supplement: Supplementary file 1 [file pathogens-15-00263-s001.zip › pathogens-4153905-supplementary.pdf]

Supplementary Materials

Supplementary Figure S1. Cell viability assay of *Acanthamoeba castellanii* trophozoites during adhesion kinetics.

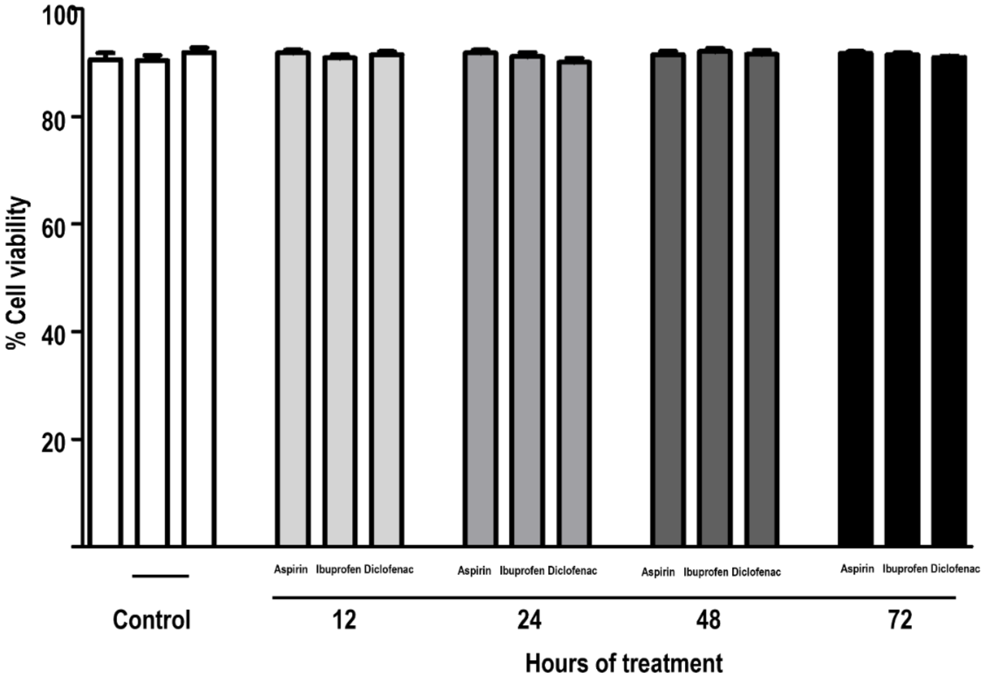

The percentage of viable trophozoites was determined by trypan blue exclusion at each time point after exposure to different NSAIDs. Data represent three independent biological replicates performed in triplicate

Supplementary Figure S2. Actin cytoskeleton organization in MDCK cells co-cultured with *Acanthamoeba castellanii* trophozoites and NSAIDs.

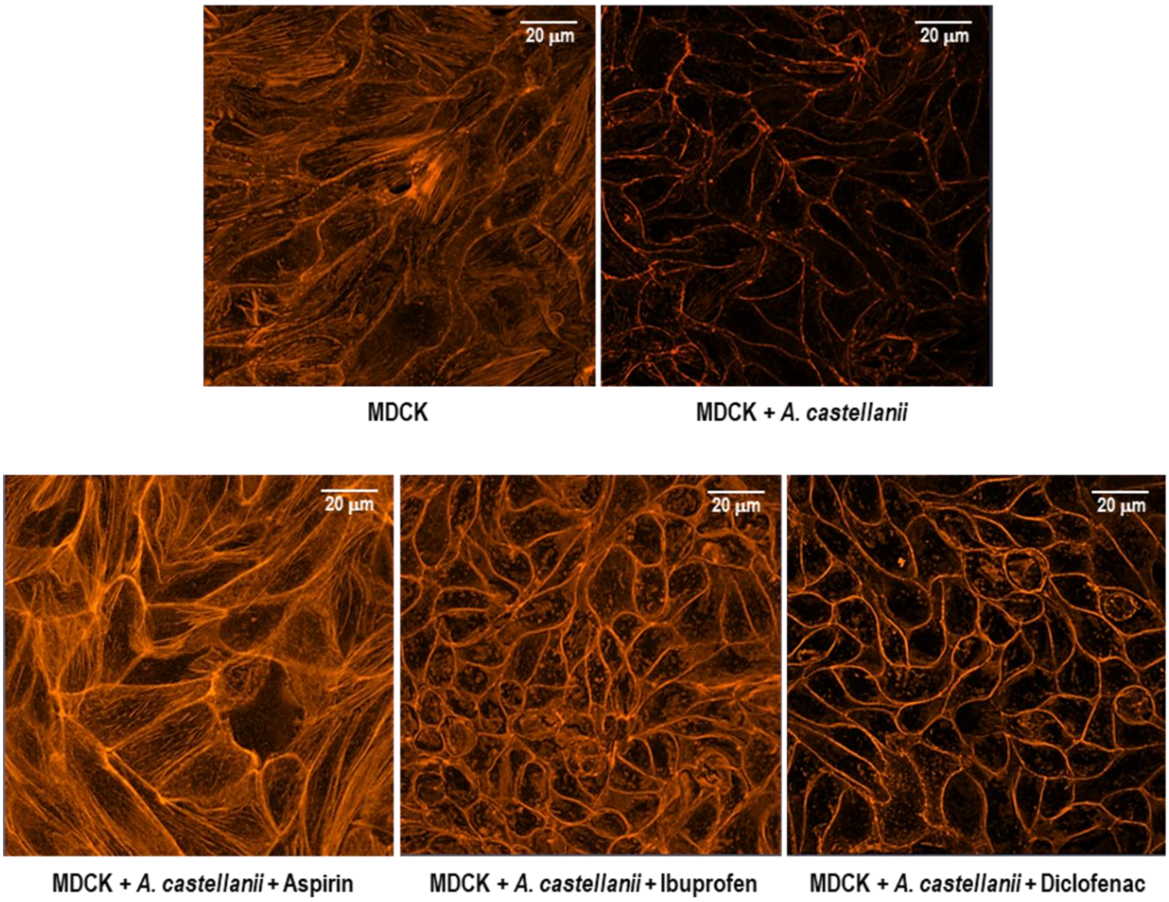

Confocal images were acquired using a Carl Zeiss LSM 900 confocal microscope equipped with a 40× oil-immersion objective (numerical aperture, NA 1.3). Actin filaments were stained with rhodamine–phalloidin. Images were processed using ZEN Black Edition software. MDCK cells were co-incubated with trophozoites and the indicated drugs for 48 h.

Supplementary figure S3: Fluorescence Measurement.

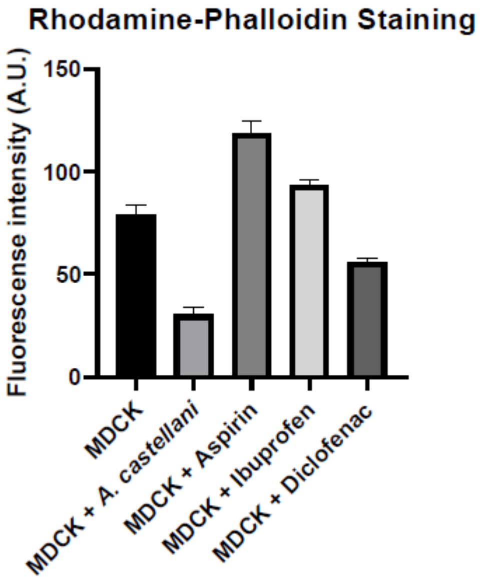

Using the Image J software, the confocal images were converted to grayscale. Then, the set measurements were established for area, mean gray value, and integrated density. The areas were selected using the drawing tool to define the regions of interest (ROI). Also, the background subtraction was performed selecting a blank region and subtracting this value from the fluorescence data for each confocal result. This process was realized in three different fields of the images from different conditions. Finally, statistical analysis was performed in GraphPad Prism software.

Supplementary Figure S4. Confocal analysis of *Acanthamoeba castellanii* cysts.

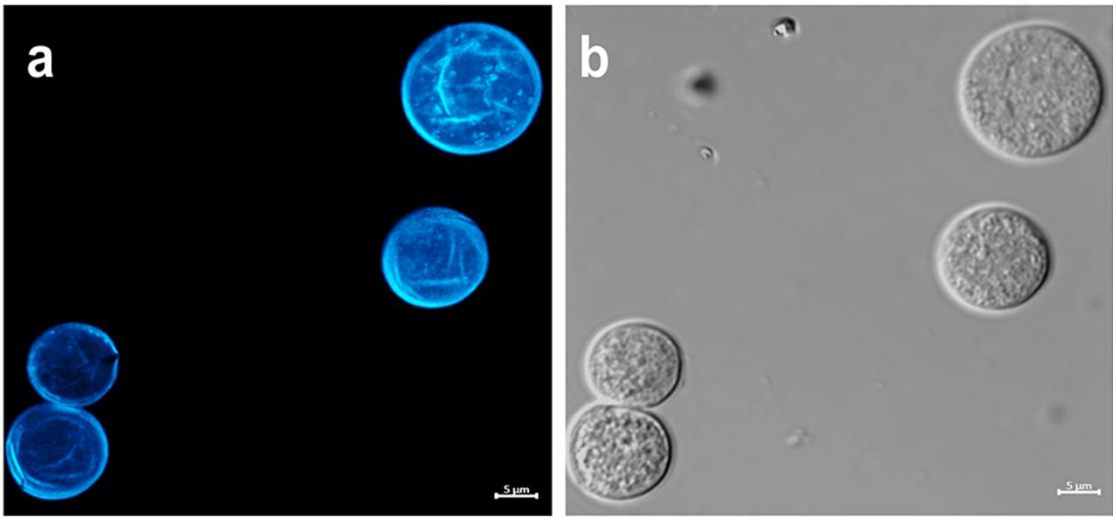

a) Cyst populations were stained with Calcofluor White and analyzed by confocal microscopy. **b)** Differential interference contrast (DIC) image of purified cysts acquired using a Carl Zeiss LSM 900 confocal microscope equipped with a 40× objective (numerical aperture, NA 1.3). Images were processed using ZEN Blue Edition software.

Supplementary Figure S5. Protein sequence alignment and predicted subcellular localization signals of gp63 metalloproteases.

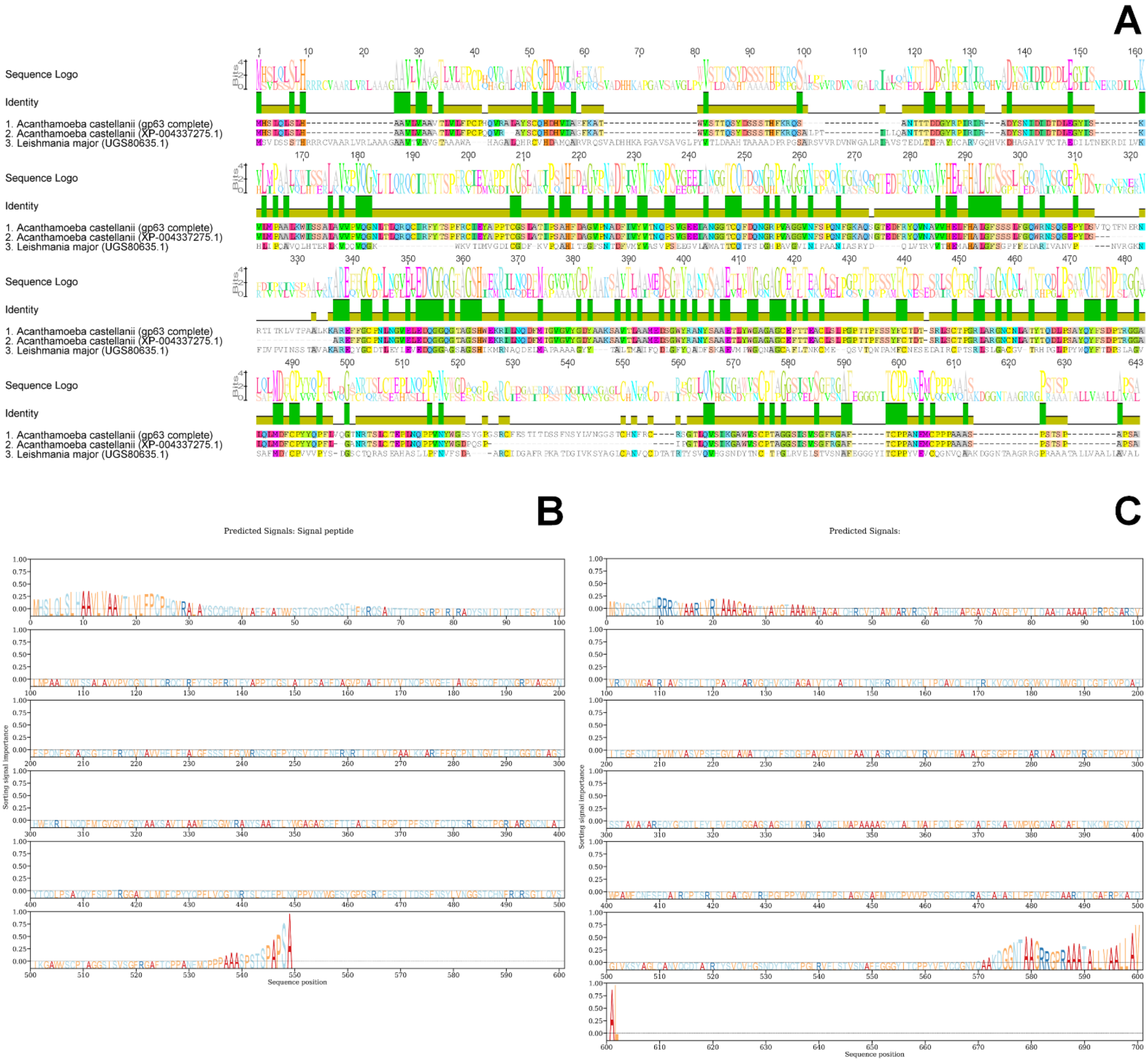

(A) Sequence alignment and sequence logo representation highlighting conserved residues and regions of structural and functional similarity between the gp63 proteins. **(B–C)** Signal peptide prediction profiles generated with DeepLoc. Probabilities for signal peptide presence are plotted across the amino acid positions of each sequence.

Supplementary Figure S6. ***In silico*** **ADME, drug-likeness, and toxicity prediction profiles of NSAIDs evaluated against** ***Acanthamoeba castellanii*****.**

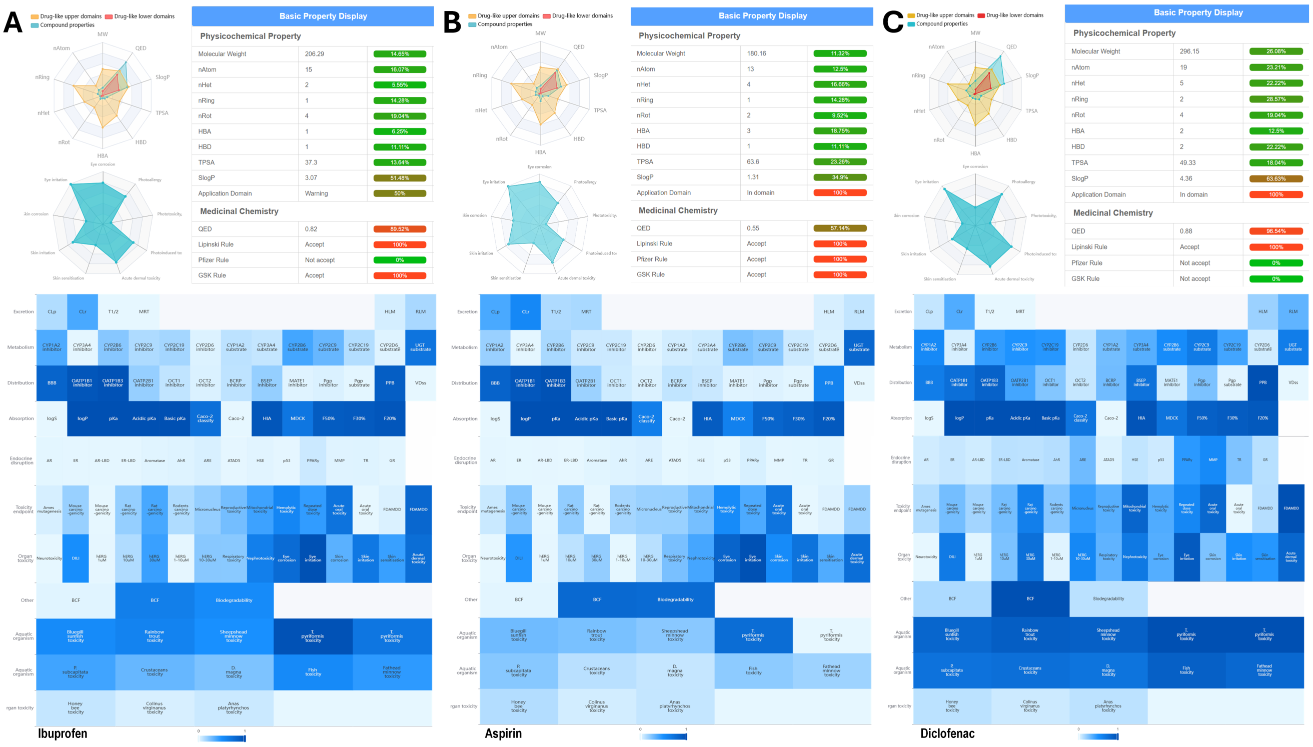

Upper Panel, Radar plots and physicochemical property summaries illustrating predicted molecular descriptors and drug-likeness parameters for ibuprofen, aspirin, and diclofenac. All compounds were predicted to comply with Lipinski's rule-of-five criteria. QED values and safety model filters (Pfizer and GlaxoSmithKline) are indicated. Down Panel, Heatmaps of predicted ADMET and toxicity-related parameters, including intestinal absorption, blood–brain barrier permeability, P-glycoprotein interaction, cytochrome P450 inhibition, hERG channel liability, benchmark dose (BMD), and cytotoxicity endpoints. Structural similarity to selected toxic analogs is also displayed (down panel). These in silico analyses may suggest compound-specific differences in pharmacological behavior and potential safety liabilities. (A) Ibuprofen, (B) aspirin, and (C) diclofenac.

Supplementary Figure S7. Structural similarity profiles of NSAIDs and selected toxic analogs.

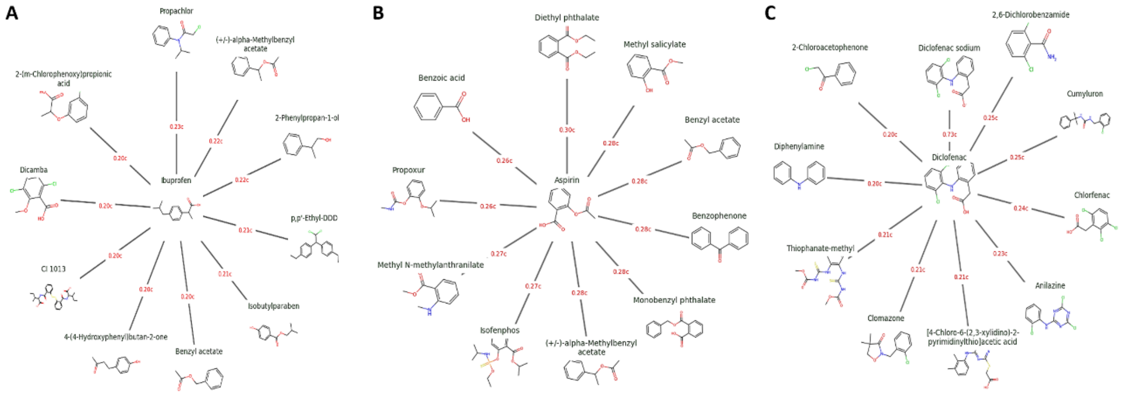

Structural similarity networks for (**A**) Ibuprofen, (**B**) Aspirin, and (**C**) Diclofenac, showing their closest toxic analogs based on computed similarity coefficients. Each panel highlights compound-specific relationships that may influence pharmacological behavior and potential safety risks. *In silico* ADME, drug-likeness, and toxicity prediction profiles of NSAIDs evaluated against *Acanthamoeba castellanii*.

Supplementary Table S1. Preparation of NSAID stock solutions, working concentrations, and vehicle controls

Compound (Brand, Manufacturer)	Pharmaceutical presentation	Stock preparation	Stock solvent	Final working concentration	Vehicle control (final concentration)
Acetylsalicylic acid (Aspirin®, Bayer)	500 mg effervescent tablets	Tablets dissolved to prepare concentrated stock solutions	Sterile distilled water	100 µM (diluted in culture medium)	Water (volume-matched)
Sodium diclofenac (AMSA)	75 mg/3 mL injectable ampoule	Commercial injectable solution used directly; diluted in medium	Aqueous injectable formulation	100 µM (direct dilution in culture medium)	Medium only
Ibuprofen (Gelubrin®)	600 mg oral capsules	Capsule contents extracted and dissolved to prepare concentrated stock solutions	Dimethyl sulfoxide (DMSO)	100 µM (diluted in culture medium)	DMSO (≤0.1% v/v)
